# Effect of sulfur on enhancing nitrogen-doping and magnetic properties of carbon nanotubes

**DOI:** 10.1186/1556-276X-6-77

**Published:** 2011-01-12

**Authors:** Tongxiang Cui, Ruitao Lv, Zheng-hong Huang, Feiyu Kang, Kunlin Wang, Dehai Wu

**Affiliations:** 1 Laboratory of Advanced Materials, Department of Materials Science and Engineering, Tsinghua University, Beijing 100084, PR China; 2 Key Laboratory for Advanced Manufacturing by Materials Processing Technology and Department of Mechanical Engineering, Tsinghua University, Beijing 100084, PR China

## Abstract

Sulfur (S) is introduced as an additive in the growth atmosphere of carbon nanotubes (CNTs) in the range of 940-1020°C. CNT products with distorted sidewalls can be obtained by S-assisted growth. Moreover, many fascinating CNT structures can also be found in samples grown with S addition, such as bamboo-like CNTs, twisted CNTs, arborization-like CNTs, and bead-like CNTs. Compared with CNTs grown without S, more nitrogen-doping content is achieved in CNTs with S addition, which is beneficial for the properties and applications of nitrogen-doped CNTs. In addition, S can also enhance the encapsulation of ferromagnetic materials and thus improve the soft magnetic properties of CNTs, which is favorable to the applications of CNTs in the electromagnetic wave-absorbing and magnetic data storage areas.

## Introduction

Sulfur (S) is an important additive for controlling the structures and properties of carbon nanotubes (CNTs). Many interesting carbon nanostructures, such as Y-junction CNTs [1], sea-urchin-like CNTs [2], long single-walled CNT (SWCNT) strands [3], double-walled CNT films [4], amorphous CNTs [5], large-diameter SWCNTs [6], ultra-short CNTs [7], and small-diameter multi-walled CNTs [8] can be obtained by S-assisted growth. Meanwhile, enhanced mechanical properties [3], water-solubility [6], catalyst support performance [6], electrochemical properties [7], and photovoltaic performance [9] can all be achieved by CNT products with S-assisted growth. Therefore, the introduction of S into growth atmosphere of CNTs has attracted much research interest because of their promotional effect on synthesis of carbon nanostructures with novel morphologies and properties.

Nitrogen-doping (N-doping) is an effective way to modify the properties of CNTs. Nitrogen-doped (N-doped) CNTs demonstrate negative differential resistance behavior [10], high electrocatalytic activity for oxygen reduction [11], and can also be used as suitable support for uniform distribution of Pt catalyst [12,13]. In addition, N-doping has a significant effect on controlling chirality and crystallinity of CNTs [14], leading to *n*-type CNTs [15], and improving the field emission performance of CNTs [16-18]. The effective doping of nitrogen into CNTs is mainly achieved *in situ *during CNT growth [12-16,19], and N-doping content is usually controlled by adjusting the ratio of carbon source to nitrogen source [16,19], which is disadvantageous for increasing the doping content of CNTs produced with liquid carbon source. For example, N-doped CNTs can be synthesized by mixture of xylene and pyridine, and CNTs with different N-doping contents are obtained from different xylene/pyridine ratios, but their doping contents cannot be larger than that with pure pyridine [19]. Therefore, it is crucial to develop an efficient way to improve the N-doping content in CNTs from the view of both theoretical and applied research.

In this study, S was introduced as an additive in the growth of CNTs. CNTs with distorted walls were obtained, which might be good supports for catalyst nanoparticles. Many other fascinating CNT structures were also obtained in this study's samples, such as bamboo-like CNTs, twisted CNTs, arborization-like CNTs, and bead-like CNTs. When S was used as growth promoter, the improved CNTs with high N-doping content could be achieved. In addition, the effect of S addition on enhanced soft magnetic properties of CNTs was also demonstrated.

## Experimental

Experimental setup and procedure are similar to that described in the previous report by the authors about N-doped CNT arrays [18]. Ferrocene and pure S powders were dissolved in acetonitrile to form a solution (20 mg/ml), and fed into chemical vapor deposition (CVD) reactor by a syringe pump at a constant rate of 0.4 ml/min for 0.5-1 h. A mixture of Ar (2000 sccm) and H_2 _(300 sccm) acts as the carrier gas. In order to investigate the effect of S on CNT growth at a relatively low temperature, the temperature is set in the range of 940-1020°C. The S concentration in the catalyst (atomic ratio of S:Fe) is 1:10. CNTs without S additive are also prepared for comparison.

The resulting CNTs were characterized using scanning electron microscope (SEM, JOEL JSM-6460 LV SEM), transmission electron microscope (TEM, JEM-200 CX), microscopic confocal Raman spectrometer (Renishow RM 2000, using 632.8-nm laser excitation), Auger electron spectroscopy (AES, PHI-700), and X-ray diffractometer (XRD, Bruker D_8 _advence). The magnetization measurements were performed on a vibrating sample magnetometer (LakeShore VSM-7307) at room temperature. Thermogravimetric analysis (TGA) results were obtained using 6-mg samples in air flow at a heating rate of 20°C/min.

## Results and discussion

**Figure 1:**
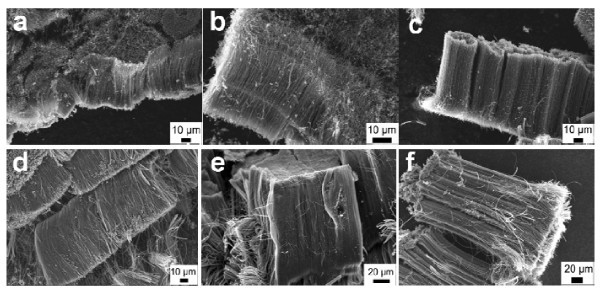
**SEM images of as-grown CNTs at three different temperatures with and without S**. (a-c). Different temperatures with S: **(a) **940°C, **(b) **980°C, **(c) **1020°C; **(d-f) **different temperatures without S: **(d) **940°C, **(e) **980°C, **(f) **1020°C.

Figure 1 shows the SEM images of as-grown CNTs at different temperatures with and without S. It can be seen from Figure 1a, b, c, d, e, f that aligned CNTs are obtained at all the three temperatures(940°C, 980°C, and 1020°C) with and without S. Aligned CNTs produced by acetonitrile without S have been reported in our previous study [18], and the results of this study demonstrate that the introduction of S is nondestructive to the alignment of CNTs.

**Figure 2:**
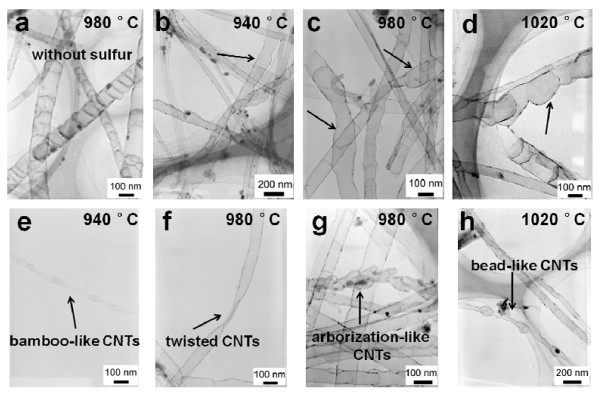
**TEM images of as-grown CNTs at three different temperatures with and without S**. **(a) **980°C without S; **(b-d) **different temperatures with S: **(b) **940°C, **(c) **980°C, **(d) **1020°C; **(e-h) **novel CNT structures with S: **(e) **bamboo-like CNTs, **(f) **twisted CNTs, **(g) **arborization-like CNTs, **(h) **bead-like CNTs.

The TEM images of the as-grown products are shown in Figure 2. Typical morphology of CNTs grown without S is shown in Figure 2a, and their sidewalls are straight as those shown in many previous reports [11,18,20]. However, in the case of products obtained by the S-assisted method, it can be clearly seen from Figure 2b, c, d that CNTs with distorted sidewalls can be obtained at different growth temperatures. Owing to the existence of abundant defect in surface, this kind of CNTs might supply more active positions for efficient immobilization of catalyst nanoparticles, and thus be useful for catalyst support applications [21]. Previous studies have demonstrated that S is favorable for the formation of pentagon and heptagon carbon rings, inducing curvature and further influencing the CNT morphology [1,2]. Many pentagonal and heptagonal rings exist in the *sp*^2 ^carbon lattice, which are supposed to cause the distort-walled CNTs formation. Furthermore, many fascinating CNT structures can also be found in the CNT samples of this study with S, such as bamboo-like CNTs (Figure 2e), twisted CNTs (Figure  2f), arborization-like CNTs (Figure 2g), and bead-like CNTs (Figure 2h).

**Figure 3:**
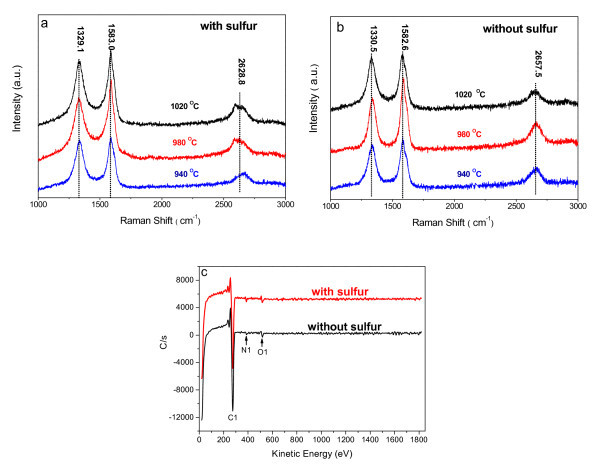
**Raman spectra and surface AES of CNTs**. **(a, b) **Raman spectra of CNTs grown at different temperatures with and without S: **(a) **with S, **(b) **without S; **(c) **Surface AES of the CNTs produced at 980°C with and without S.

Raman spectroscopy has been proved to be a perfect tool to evaluate the crystallinity and defects in carbon structures [18,20]. The Raman spectra of the CNTs grown at different temperatures with and without S are shown in Figure 3a, b. The strong bands around 1330, 1580, and 2650 cm^-1 ^can be assigned to D-band, G-band, and 2D-band, respectively. Taking D- and G-bands into consideration, little difference is found between the CNTs with and without S. The obvious difference between their 2D-bands is shown in Figure 3. If S is introduced into the CVD reactor, then 2D-band downshifts approximately 30 cm^-1^. This might be attributed to the improved N-doping content [22]. The 2D-band of CNTs without S is almost symmetrical, but 2D-band is irregular with S. This may be due to the difference between their CNT walls, because 2D-band is sensitive to the stacking of graphene sheets [23].

The improvement of N-doping content is also confirmed by AES. Figure 3c shows the surface AES results of the two CNTs samples produced at 980°C with S and without S, respectively. Almost no signals of Fe were detected in both of the samples, indicating that Fe catalyst particles are fully covered by carbon layers. The CNTs produced with S consist of C (95.2 at.%), N (2.4 at.%), and O (2.4 at.%), while CNTs without S consist of C (96.9 at.%), N (1.2 at.%), and O (1.9 at.%). The presence of O can be attributed to the exposure of the CNTs in the air atmosphere [18,24]. It can be seen that S has an effect on increasing N-doping content in CNTs. This might be because S induces pentagon and heptagon in *sp*^2 ^carbon lattice [1,2], and these heterocyclic rings are supposed to be favorable to the enhancement of N-doping.

**Figure 4:**
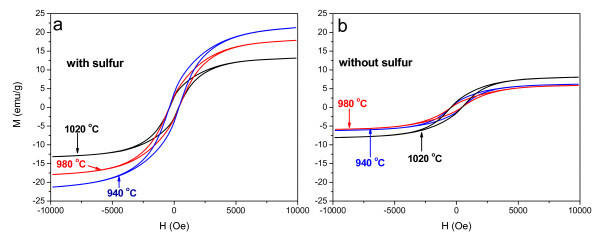
**Hysteresis loops of CNTs produced at different temperatures with and without S: (a) with S, (b) without S**.

Hysteresis loops and the saturation magnetization (*M*_s_) of CNTs produced at different temperatures with and without S are shown in Figure 4 and Table 1, respectively. As shown in Table 1, the *M*_s _of CNTs with S is enhanced by 1.5-3.5 times compared with that without S. As proved in our previous study, the *M*_s _improvement of CNTs can be attributed to the increased encapsulation of ferromagnetic materials into their inner cavities [25]. Conversely, the enhanced *M*_s _also indicates that S can increase the encapsulation of ferromagnetic materials into CNTs.

**Table 1:** The saturation magnetization (*M*_s_, emu/g) of CNTs produced at different conditions

Temperature (°C)	940	980	1020
With S	21.2	17.9	13.1
Without S	5.9	6.2	7.9

**Figure 5:**
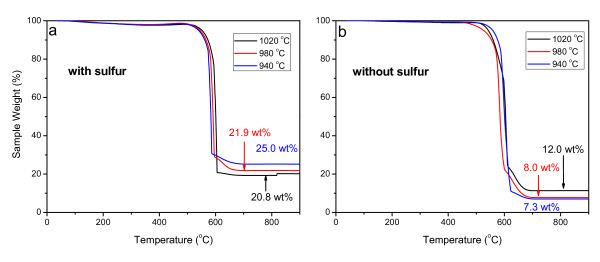
**TGA curves of CNTs produced at different temperatures with and without S: (a) with S, (b) without S**.

In order to prove the enhanced encapsulation of ferromagnetic material, TGA was carried out, as shown in Figure 5. The residual weight percentage in the platform part of TGA curves at 600-900°C range can be used to determine the content of ferromagnetic material inside the CNTs [26]. It can be seen that the introduction of S can remarkably increase the encapsulation of ferromagnetic material compared with that without S.

**Figure 6:**
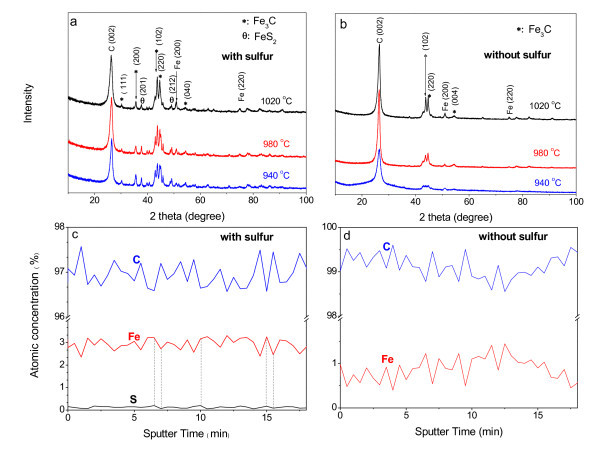
**XRD and Auger depth scanning spectra of CNTs**. **(a, b) **XRD spectra of CNTs produced at different temperatures with and without S: **(a) **with S, **(b) **without S; **(c, d) **Auger depth scanning spectra of CNTs produced at 980°C with and without S: **(c) **with S, **(d) **without S.

The XRD analysis proved the existence of pure Fe and FeC_3 _in the CNTs grown with and without S, as shown in Figure 6a, b. Auger depth-resolved chemical analysis was carried out by sputtering the catalyst particle seeking with SEM using an *in situ *1 keV Ar-ion gun, (sputtering speed: 13.5 nm/min, probe diameter 1.8 nm), and the resulting Auger depth profiles of CNTs grown at 980°C with and without S are shown in Figure 6c, d. The major difference between Figure 6c, d is that S peak exists in Figure 6c. This indicates the existence of iron S compound in CNTs produced with S, which is in accordance with the XRD result (Figure 6a). CNTs are good templates for housing metals or compounds, and these nanostructures combine the properties of metals/compounds and CNTs together [25]. S can enrich the categories and enhance the amount of encapsulation in CNTs, which is favorable to the applications of CNTs in the electromagnetic wave-absorbing [25], and magnetic data storage [27] areas.

## Conclusions

CNTs with distorted sidewalls are synthesized by the S-assisted CVD method. S is favorable to the formation of pentagon and heptagon carbon rings, and these rings are supposed to result in the formation of CNTs with distorted sidewalls. This kind of CNTs might supply more active positions for catalyst nanoparticles and have potential usage in catalyst support area. It is also found that S addition can enhance the N-doping content of CNTs, resulting from heterocyclic rings, which are favorable for the enhancement of N-doping. The effect of S on enhancing the soft magnetic properties of CNTs is also demonstrated, and the enhanced saturation magnetization can be attributed to the enhanced encapsulation of ferromagnetic material. Enhanced soft magnetic properties of CNTs are favorable for the applications of CNTs in the electromagnetic wave-absorbing and magnetic data storage areas.

## Abbreviations

AES: Auger electron spectroscopy; CNTs: carbon nanotubes; CVD: chemical vapor deposition; N: nitrogen; S: sulfur; SEM: scanning electron microscope; SWCNT: single-walled CNT; TGA: thermogravimetric analysis; TEM: transmission electron microscope; XRD: X-ray diffraction.

## Competing interests

The authors declare that they have no competing interests.

## Authors' contributions

TC carried out the most of experiments and drafted the manuscript. RL participated in the CNT synthesis and manuscript preparation. ZH participated in the analysis of Raman and XRD spectra. FK designed the experiments and revised the manuscript. KW and DW discussed and analyzed the experimental results. All authors read and approved the final manuscript.
